# Molecular cloning and comparative sequence analysis of fungal β-Xylosidases

**DOI:** 10.1186/s13568-016-0202-3

**Published:** 2016-04-14

**Authors:** Ghulam Mustafa, Sumaira Kousar, Muhammad Ibrahim Rajoka, Amer Jamil

**Affiliations:** 1 Department of Biochemistry, University of Agriculture, Faisalabad, 38040 Pakistan; 2 Department of Chemistry, Government College Women University, Faisalabad, Pakistan; 3 Department of Bioinformatics and Biotechnology, Government College University, Faisalabad, Pakistan

**Keywords:** Hemicellulose, Exoglycosidase, Xylanolytic enzymes, β-Xylosidase, β-Xylosidase cloning, β-Xylosidase expression

## Abstract

Commercial scale degradation of hemicelluloses into easily accessible sugar residues is practically crucial in industrial as well as biochemical processes. Xylanolytic enzymes have a great number of possible applications in many biotechnological processes and therefore, these enzymes are continuously attracting the attention of scientists. Due to this fact, different β-Xylosidases have been isolated, purified and characterized from several bacteria and fungi. Microorganisms in this respect have gained much momentum for production of these significant biocatalysts with remarkable features. It is difficult to propagate microorganisms for efficient and cost-competitive production of β-Xylosidase from hemicelluloses due to expensive conditions of fermentation. The screening of new organisms with an enhanced production of β-Xylosidases has been made possible with the help of recombinant DNA technology. β-Xylosidase genes haven been cloned and expressed on large scale in both homologous and heterologous hosts with the advent of genetic engineering. Therefore, we have reviewed the literature regarding cloning of β-Xylosidase genes into various hosts for their heterologous production along with sequence similarities among different β-Xylosidases. The study provides insight into the current status of cloning, expression and sequence analysis of β-Xylosidases for industrial applications.

## Introduction

Microorganisms are the natural producers of enzymes due to their abundance in nature and are thus central to biomass conversion. Biomass consists of cellulose as a primary component while hemicellulose being the second most abundant constituent of plant cell wall comprises a heterogeneous molecule named xylan (Ahmed et al. 2012). Microorganisms efficiently hydrolyse xylan into monosaccharides through the action of a battery of enzymes to obtain higher yields for industrial applications. Complete degradation of xylan backbone into its monomers by microbial world is a multistep process which requires concerted action of hemicellulases and cellulases based cocktail. Xylanases and β-Xylosidases are among the major enzymes of this cocktail (Menon et al. 2010).

**Fig. 1:**
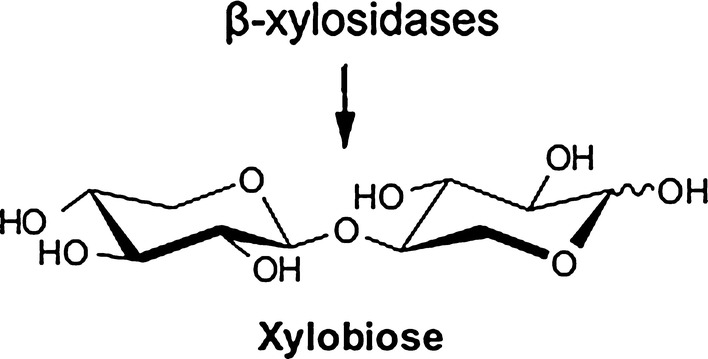
Schematic representation of hemicellulose degradation by β-Xylosidases which release xylose from xylobiose (Kousar et al. 2013)

β-Xylosidase (EC 3.2.1.37) is an exoglycosidase having ability to hydrolyse the non-reducing ends of xylooligosaccharides into xylose (Saleem et al. 2012) (Fig. 1). It is widely disseminated in nature and is one of the component enzymes of hemicellulose complex (Rajoka 2007). For large scale production of β-Xylosidase, microbial cultivation is very unwieldy and it often ends up in many interfering enzymes as a pure form of a particular enzyme becomes difficult to be isolated from a microbial preparation. Therefore, efficient and cost effective hydrolysis of biomass is generally preferred and alternative sources are being searched (Kanna et al. 2011). In this respect fungi and bacteria play a central role in the degradation of complex plant polymers. This hydrolysis is accomplished by the xylan hydrolyzing enzymes produced with novel and desirable characteristics (Banerjee et al. 2010). Filamentous fungi and mesophilic and thermophilic bacteria are considered to be the attractive source of β-Xylosidase (Lorenz and Wiegel 1997). However, productivity and stability varies among different microbial species with respect to practical applications. Therefore, natural sources for the enzyme with increased thermostability, greater specific activity, efficient ability of translation and more resistance to proteases are of considerable interest for industrial applications.

Before recombinant DNA technology the industrially important enzymes were produced from microorganisms. Genetic engineering has been proved to be an alternative to the extreme and expensive conditions of fermentation. In spite of the availability of biochemical and molecular information about β-Xylosidase a very little progress at the genetic level has been reported. A detailed evaluation of the biochemical characterization and regulation of the enzyme at molecular level requires genetic analysis of the gene encoding β-Xylosidase (Girio et al. 2010). In our previous work we discussed a detailed study of biochemical characterization of β-Xylosidase from bacterial and fungal sources (Kousar et al. 2013) whereas here we report gene manipulation of the enzyme and its expression in different hosts.

## Cloning of fungal β-Xylosidase genes

Fungi have been used for more than 50 years for the production of industrially important enzymes and microbial biomass proteins (Mustafa and Jamil 2013). From a pool of fungal proteins, the isolation of required enzymes requires not only monotonous steps but also increases their costs (Montibeller et al. 2014). For this purpose, recombinant DNA technology is used with more success as these enzymes can be expressed in both homologous and heterologous protein expression hosts on a large scale (Korona et al. 2006). For enhanced production of industrially important enzymes by improved substrate utilization and other useful properties on commercial scale, many genes have been cloned and expressed (Ahmed et al. 2009). The selection of xylanolytic enzymes having industrial applications is a result of recombinant DNA technology (Kvesitadze et al. 2014).

From *Aspergillus niger* a 1.9 kb DNA fragment containing β-Xylosidase gene (*xlnD*) was generated and used as a specific probe (Perez-Gonzalez et al. 1998). A complete β-Xylosidase gene (*xloA*) was isolated from *Vibrio* sp. strain XY-214 genomic DNA with the help of AlkPhos-labeled probe that gave 4.2 kbp fragment of *xloA* (Umemoto et al. 2008). The coding region of β-Xylosidase (*xlnD*) gene from *A. niger* yielded a mature protein of 85.3 kDa (Perez-Gonzalez et al. 1998) that resembles closely to the β-Xylosidase previously isolated from *A. nidulans* having a molecular mass of 85 kDa (Kumar and Ramon 1996). The activity and thermostability of β-Xylosidase and xylanase from *A. ochraceus* have been improved by mutagenesis through UV and NTG (Biswas et al. 1990).

### Cloning in *Fusarium* sp

So far most of the studies on genetic manipulation of *Fusarium* sp. have been carried out related to phytopathological action of *Fusarium oxysporum*. First homologous transformation system of this fungus was developed by (Diolez et al. 1993) that was based on cloning of *nia* gene encoding NR from *F. oxysporum*. Gene replacement events, integrations at non-homologous sites and single-copy homologous integrations were observed, and transformation frequencies were achieved up to 6000 transformants per µg of DNA. The transformation system in *F. oxysporum* was offered by new applications of gene replacement events which were observed at high frequency (in 50 % of the transformants). Gareia-Pedrajas and Roncero (1996) reported a highly efficient transformation system in *F. oxysporum* including DNA transformation of protoplasts mediated by PEG and protoplasting based on a mutant of NR which was complementary to homologous *nitl* gene and on the autonomously replicating sequence (ARS) and telomeric sequences presence in the vector. The applications of *Agrobacterium tumefaciens*-mediated transformation (ATMT) to *F. oxysporum* were described by Mullins et al. (2001) through novel binary vectors constructions, selection of ATMT conditions that affect the efficiency of transformation, inserted T-DNA copy number in *F. oxysporum* and bringing hygromycin B phosphotransferase gene (*hph*) from bacteria under *A. nidulans* promoter (trpC) as a selectable marker.

A gene replacement method in *F. oxysporum* was reported by Khang et al. (2005) based on ATMT with a mutant allele of targeted gene and as a marker of conditional negative selection against ectopic transformants. The gene was flanked by *HSVtk* gene having the function to convert 5-fluoro-2′-deoxyuridine into a compound that is toxic to various fungi. The mutants of gene replacement lack HSVtk while it is expressed by ectopic transformants. Through counter-selection against ectopic transformants identification of targeted mutants is facilitated by growing transformants on a medium supplemented with 5-fluoro-2′-deoxyuridine. Following the treatments with UV or NTG the activity of β-Xylosidase and xylanase enzymes was enhanced by more than threefold through mutagenesis of *F. oxysporum* DSM 841 (Singh et al. 1995).

### Cloning in *Escherichia coli*

*Escherichia coli* is an ideal platform for the expression of recombinant proteins (Tables 1, 2). There are heterologous proteins such as xylanases which cannot be expressed in this host functionally due to repetitive appearance of rare codons in addition to disulfide bond formation (Stewart et al. 1998) and glycosylation like requirements for specific post translational modifications. *E. coli* can perform simple O-glycosylation only but xylanases require N-glycosylation (Messner 2009). Exceptionally, from a thermophilic fungus *P. thermophila* a glycosylated β-Xylosidase gene was functionally expressed in *E. coli* (Teng et al. 2011). The extracellular expression of this gene has indicated that glycosylation was not essential for its activity. From *Klebsiella oxytoca* a xylo-oligosaccharide (XOS) utilization operon encoding a xylosidase and xylobiose/cation symporter was expressed in *E. coli* KO11. This expression resulted in successful consumption of XOS having xylosyl up to six residues (Qian et al. 2003). Therefore, multiple factors including rapid growth on inexpensive media, simple practices of transformation and easy isolation and purification techniques have made *E. coli* a successful candidate of recombinant expression platform.

**Table 1:** Cloning of different bacterial β-Xylosidase genes in *E. coli*

Source organism	Gene	Vector	Host	Molecular mass of the recombinant enzyme (kDa)	Characteristics of the recombinant enzyme	Reference
*C. stercorarium* F-9	*Xyl43B*	pYK306, pT7Blue T, pET-28a	*E. coli* JM109, *E. coli* DH5α	56.3	Temperature = 80 °C pH 3.5 enzyme activity = 10 U mg^ − 1^stability 15 min at 50–70 °C K_m_ = 6.2 mm V_max_ = 15 μmol min^−1^ mg^−1^	Suryani et al. (2004)
*C. stercorarium* F-9		pBR322	*E. coli* JM109	–	Temperature = 80 °C enzyme activity = 8.16 U mg^ −1^	Sakka et al. (1990)
*Butyrivibrio fibrisolvens* GS113	*xylB*	pUC18	*E. coli* DH5α	60	Specific activity 0.2 nmol min^−1^ mg^−1^	Utt et al. (1991)
*B. stearothermophilus* 21	*xylA*	pUC19	*E. coli* JM109	75	Enzyme activity 1.56 U 10 mL^−1^	Baba et al. (1994)
*Thermoanaerobacterium* sp. JW/SL YS485	*xylB*	pUC18	*E. coli* TG-1	58.5	Temperature = 65 °C pH = 6.0 specific activity = 0.53 U mg^ −1^	Lorenz and Wiegel (1997)
*Thermotoga thermarum* DSM 5069	*XynB3*	pET-20b	*E. coli* Top10 and BL21 (DE3)	85 kD	Temperature = 95 °C pH 6.0 specific activity = 116 U mg ^−1^Km = 0.27 mM V_max_ = 223.2 U mg^−^^1^ K_cat_/K_m_ 1173.4 mM^−1^ s^−1^	Shi et al. (2013)
*P. ruminicola* B_1_4	*xynB*	pUC19/L3	*E. coli* DH 5α	–	pH = 6.5 enzyme activity = 540 nmol min^−1^ mg^−1^	Gasparic et al. (1995)
*C. saccharolyticum* Tp8T6.3.3.1	*aryl*	pBR322	*E.coli*	53	Temperature = 70 °C pH = 5.7 specific activity = 49.2 µmol min^−1^ mg^−1 km^ = 10 mM V_max_ = 64 U mg^−1^	Hudson et al. (1991)
*Bifidobacterium breve* K-110	*XylBK*	pGEM-T	*E. coli* BL21(DE3) JM109(DE3)	55.7	Temperature = 45 °C pH = 6.0 specific activity = 3.32 Umg^−1 km^ = 1.45 mM V_max_ = 10.75 μmol min^−1^ mg^−1^	Hyun et al. (2012)
*Thermobifida fusca*	*Xyl43A*	pIJ702	*E. coli* BL21(DE3)/pLysS	–	Temperature = 55–60 °C pH = 5.5–6.0 km = 0.23 mM K_cat_ = 4.83 s^−1^	Morais et al. (2011)
*Ruminococcus albus* 8	*Xyl3A*	pET-46b	*E. coli* JM109	77.3		Moon et al. (2011)
*S. thermoviolaceus* OPC-520	*bxlA*	pUC18 pUC19	*E. coli* JM109	82	Optimum temperature = 50 °C optimum pH = 6.5 stable at 50 °C activity lost at 60 °C	Tsujibo et al. (2001)
*Thermoanaerobacterium saccharolyticum* B6A-RI	*xynB*	pHC79 (BRL)	*E. coli* DH5a	60	Optimum temperature = 65 °C loss of activity at 85 °C half-life = 55 min at 75 °C	Lee and Zeikus (1993)
*Thermoanaerobacterium saccharolyticum* JW/SL-YS485	*XylC*	pMD19-T	*E. coli* JM109	78	Temperature = 65 °C pH = 6.0 specific activity = 45.8 U mg^−1^ half-life = 1 h at 67 °C K_m_ = 28 mM V_max_ = 276 U mg^−1^	Shao et al. ( 2011)
*P. woosongensis* KCTC 3953 (DSM 16971)	*XylC*	pUC19 pET23a(+)	*E. coli* DH5α *E. coli* BL21(DE3)	55	Temperature = 95 °C pH = 6.5 specific activity = 659.9 mU mg−1 protein K_m_ = 8.5 mM V_max_ = 3.1 µmol min^−1^mg^−1^ half-life = 22 min at 95 °C	Kim and Yoon (2010)
*Butyrivibrio fibrisolvens* GS113	*xylB*	pUC18	*E. coli* DHS5α	60	Temperature = 60 °C pH = 5.5 specific activity = 0.13 µmol min^−1^ mg^−1^	Sewell et al. (1989)
*B. halodurans* C-125	*Bxyl*	pQEBxyl	*E coli* JM109	61	Temperature = 45 °C pH = 7.0 enzyme activity = 7811.1 mU K_m_ = 1.9 mmol L^−1^ V_max_ = 0.65 µmol min^−1^ mg^−1^	Liang et al. (2009)
*Vibrio* sp. XY-214	*xloA*	pBluescriptII KS (−)	*E. coli* DH5α	60	Temperautre = 35 °C pH = 7.0 km = 0.244 mM V_max_ = 1.82 µmol min-1 mg^−1^	Umemoto et al. (2008)

**Table 2:** Cloning of different fungal β-Xylosidase genes in *E. coli*

Source organism	Gene	Vector	Host	Molecular mass of the recombinant enzyme (kDa)	Characteristics of the recombinant enzyme	Reference
*Acremonium cellulolyticus* Y-94	*bxy3A*	pLD10	*E. coli* DH 5α		Enzyme activity = 14.75 U L^−1^	Kanna et al. (2011)
*T. reesei* RutC-30	*bxl1*	pAJ401	E.coli JS4	49.1	Enzyme activity = 16.3 nkat mL^−1^	Margolles-Clark et al. (1996)
*A. niger* 90,196	*xlnD*	pGEM-T Easy	*E. coli* XL1-Blue MRF	85.1	Temperature = 60 °C pH = 3.2 enzyme activity = 5.3 nkat mL^−1^ stability = 50 °C	Grange et al. (2001)
*A. nidulans* G191	*xlnD*	pGW635	*E. coli* LE392 *E. coli* DH5α	85		Perez-Gonzalez et al. (1998)
*A. niger* GS1	*xlnD*	pAN52.1	*E. coli* JM109	90	Temperature = 70 °C pH = 3.6 enzyme activity = 4280 Umg protein^−1^ half-life = 74 min at 70 °C Activation energy = 58.9 kJ mol^−1^	Amaro-Reyes et al. (2011)
*A. oryzae* RIB40	*xylB*	pET32b	*E. coli* DH5α, Rosetta-gami™ (DE3) pLysS	37.4	Temperature = 30 °C pH = 7 specific activity = 6.1 U/mg K_m_ = 0.48 mM V_max_ = 42.6 μmol min^−1^ mg^−1^	Suzuki et al. (2010)
*H. insolens* Y1	*Xyl43A*	pEASY-T3	*E. coli* Trans1-T1	37	Temperature = 50 °C pH = 6.5 specific activity = 20.5 U mg^−1^K_m_ = 12.2 mM V_max_ = 203.8 μmol min^−1^ mg^−1^	Yang et al. (2014)
*H. insolens* Y1	*Xyl43B*	pEASY-T3	*E. coli* Trans1-T1	62	Temperature = 50 °C pH = 7 specific activity = 1.7 U/mg K_m_ = 1.29 mM V_max_ = 2.18 μmol min^−1^ mg^−1^	Yang et al. (2014)
*T. lanuginosus* CAU44	*TlXyl43*	pET28a(+)	*E. coli* BL21 (DE3)	51.6	Temperature = 55 °C pH = 6.5 specific activity = 45.4 U mg^−1^K_m_ = 3.9 mM V_max_ = 107.6 μmol min^−1^ mg^−1^	Chen et al. (2012a)
*P. thermophile* J18	*PtXyl43*	pMD-18 T	*E. coli* BL21 (DE3)	52.3	Temperature = 55 °C pH = 7 specific activity = 45.4 U mg^−1^ K_m_ = 4.5 mM V_max_ = 90.2 μmol min^−1^ mg^−1^	Teng et al. (2011)

## Expression of β-Xylosidase genes

### Expression in *Escherichia coli*

*Escherichia coli* has been extensively used as a host microorganism for the production of recombinant proteins because of many expression advantages including simplified downstream processing, increased biological activity, enhanced product stability and solubility and authenticity of N-terminus of expressed proteins (Mergulhao et al. 2005). Considerable attention has been given to extracellular production of recombinant enzymes in *E. coli* as it has substantial advantages over periplasmic or cytoplasmic productions (Sommer et al. 2010). For heterologous gene expression in *E. coli* a number of different expression vectors and host strains are used. Liang et al. (2009) cloned *Bxyl* gene from *Bacillus halodurans* C-125 with its own promoter and transcription terminator into pQE80L expression vector and transformed into *E. coli* JM109. Using *p*-nitrophenyl-β-Xylose (pNPX) as a substrate the crude enzyme was analyzed for its xylosidase activity which was estimated to be 11 U from 450 mL cultivated cells of *E. coli*. The open reading frame of another β-Xylosidase gene (*xysB*) along with its promoter region was isolated from *Aeromonas caviae* ME-1 and cloned into pT7-Blue vector to express in *E. coli* DH5α (Suzuki et al. 2001). The specific activity of the enzyme XysB was finally enhanced to 56 nKat/mg protein.

For the expression of recombinant proteins in *E. coli* the pET expression vector systems are categorized among the most effective ones. Tsujibo et al. (2001) used an expression vector pET-20b(+) to overexpress a β-Xylosidase gene (*bxlA*) with no typical −10 and −35 promoter boxes from *Streptomyces thermoviolaceus* in *E. coli* BL21 (DE3) pLysE. The enzyme BxlA was produced in the cytoplasm induced by IPTG and from a 1.2 L culture of *E. coli* 50 mg of purified enzyme was obtained. A fungal β-Xylosidase gene *xylB* from *A. oryzae* was cloned into *Nco*I/*Xho*I site of vector pET32b and transformed in *E. coli* Rosetta-gami™ (DE3) pLysS. The enzyme was expressed with three tags i.e. *N*-terminal thioredoxin tag, His-tag and S-tag (Suzuki et al. 2010). Another β-Xylosidase gene *TlXyl43* from *Thermomyces lanuginosus* was amplified and cloned into the *Nhe*I/*Sac*I site of pET28a(+) vector and expressed in *E. coli* BL21 (DE3) (Chen et al. 2012a). Twenty-three amino acids encoded by the vector were added at the *N*-terminal of wild-type enzyme but no potential secretion signal peptides were predicted in wild-type or recombinant TlXyl43 enzyme. However, secretory expression was predicted in both wild-type and recombinant enzymes and this type of secretion should be attributed to non-classical secretion of the enzyme.

### Expression in fungi

Bacterial enzymes are mostly expressed heterologously in *E. coli* (Teng et al. 2011) but fungal enzymes are generally purified from their original cultures (Ohta et al. 2010) or produced in different high-level expression systems such as *Aspergillus* (Kitamoto et al. 1999) or *P. pastoris* (Chen et al. 2012b). With methanol induction a β-Xylosidase (Xyl3A) from *Humicola insolens* Y1 was successfully overexpressed in *P. pastoris* GS115 without signal peptide (Xia et al. 2015) and the secretion of enzyme was facilitated by using the signal peptide of the yeast α-factor into the culture supernatant. The yield of Xyl3A was found approximately 100 mg/L which suggested that it had great potential for cost effective production on a large scale. A considerable stability was found in β-Xylosidase isolated from *Paecilomyces thermophile* and expressed in *Pichia pastoris*. After 72 h of incubation the enzyme was found with maintained activity at optimal temperature of 60 °C and the molecular weight of enzyme was calculated to be 52.3 kDa which was found to be active at pH 7 (Juturu and Wu 2013). Another β-Xylosidase gene i.e. *Tlxyn1* was isolated from *T. lanuginosus* SSBP and cloned and expressed in *P. pastoris* GS115. A GAP promoter system executed the expression of recombinant β-Xylosidase. The coding region of *Tlxyn1* was not interrupted by any introns similar to some other β-Xylosidase genes observed from filamentous fungi (Gramany et al. 2015).

### β-Xylosidase expression system of filamentous fungi

In industry, enzyme production by fermentation using fungal expression systems has a long history. For *T. reesei* a number of genetic tools have been developed such as different transformation strategies like protoplast based transformation (Gruber et al. 1990), biolistic transformation (Te’o et al. 2002) and ARS (Zhong et al. 2006) and were shown to be successful. There are different selected markers like benomyl (Peterbauer et al. 1992; Schuster et al. 2007) and hygromycin (Mach et al. 1994) resistant, the auxotrophic markers like *hxkl* (Guangtao et al. 2010) and *pyr4* (Gruber et al. 1990), and *amdS* gene from *A. nidulans* that confers the ability to grow on acetamide as a sole source of nitrogen (Penttila et al. 1987).

Moreover, a sexual cycle in *T. reesei* (Guangtao et al. 2009) has been discovered which has further increased the industrial potential of the fungus. It was further reported that *T. reesei* could survive up to 13 days under anaerobic conditions. All these observations are in accordance with the fact that in *T. reesei* genome all genes are required for conversion of cellulosic sugars into ethanol (http://genome.jgipsf.org/Trire2/Trire2.home.html). For β-Xylosidases production, *Penicillia* have been described to be good producers (Curotto et al. 1994). Two β-Xylosidases produced from the cell surface of *P. herquei* were identified and purified (Ito et al. 2003). Different carbon sources can be used to induce and express β-Xylosidases from *Penicillia* (Krogh et al. 2004). The production of β-Xylosidases has been explored in a number of *Penicillia* species including *P. brasilianum* (Thygesen et al. 2003), *P. chermisinum* (Reese et al. 1973), *P. funiculosum* (Krogh et al. 2004), *P. herquei* (Ito et al. 2003), *P. islandicum* (Reese et al. 1973), *P. janthinellum* (Curotto et al. 1994), *P. persicinum* (Krogh et al. 2004), *P. pusillum* (Reese et al. 1973), *P. roseopurpureum* (Reese et al. 1973), *Penicillium* sp. AHT-1 (Rahman et al. 2003) and *P. wortmanni* (Reese et al. 1973). The best carbon source was found to be xylan in all these *Penicillium* species except *P. brasilianum* that was grown best on wet-oxidized wheat straw (Thygesen et al. 2003).

### β-Xylosidase expression system of yeasts

For the expression of heterologous proteins, yeasts are a good choice and preferred over bacterial expression systems (Table 3). The ability to accomplish post-translational modifications in eukaryotes, ability to grow to very high cell densities and the ability of protein secretions into fermentation media are the additional benefits of yeast expression systems. Moreover, the applications of yeasts in food industry are also accredited them with the generally recognized as safe (GRAS) status as they are free of toxins. For heterologous protein expression *Saccharomyces cerevisiae*, *Kluyveromyces lactis*, *Hansenula polymorpha*, *P. pastoris* and *Yarrowia lipolytica* are commonly used (Buckholz and Gleeson 1991). The host *S. cerevisiae* (Baker’s yeast) has been a good choice for researchers to express recombinant proteins. In 1981 for the first time the expression of recombinant proteins in *S. cerevisiae* was described but its applications as a host for expression of heterologous proteins in this yeast has diminished due to some limitations. Fermentative mode of growth, instability of recombinant plasmid DNA, hyperglycosylation of secreted proteins and retention of proteins within periplasmic space are the limitations that decrease yields of expressed proteins (Buckholz and Gleeson 1991).

**Table 3:** Cloning of different fungal β-Xylosidase genes in fungi

Source of gene	Gene	Vector	Host	Molecular mass of the recombinant enzyme (kDa)	Characteristics of the recombinant enzyme	Reference
*A. niger* GS1	*xlnD*	pGEM-T	*A. niger* AB4.1	90	Temperature = 70 °C pH = 3.6 enzyme activity = 4280 Umg protein^−1^ half-life = 74 min at 70 °C activation energy = 58.9 kJ mol^−1^	Amaro-Reyes et al. (2011)
*T. reesei* RutC-30	*bxl1*	pAJ401	*S. cerevisiae* DBY746	49.1	Enzyme activity = 16.3 nkat mL^−1^	Margolles-Clark et al. (1996)
*A. niger* 90,196	*xlnD*	pDF1	*S. cerevisiae* Y294	85.1	Temperature = 60 °C pH = 3.2 stability = 50 °C enzyme activity = 5.3 nkat mL^−1^	Grange et al. (2001)
*A. japonicas* MU-2	*xylA*	pPIC9	*P. pastoris* GS115	113.2	Temperature = 70 °C pH = 4.0 specific activity = 112 U mg^−1 km^ = 0.314 mM Vmax = 114 µmol mg^−1^ min^−1^ stability 60 °C for 30 min	Wakiyama et al. (2008)
*H. insolens* Y1	*xyl3A*	pPIC9	*P. pastoris* GS115	83.2	Temperature = 60 °C pH = 6 specific activity = 11.6 U mg^−1^ K_m_ = 2.51 mM V_max_ = 37.33 μmol min^−1^ mg^−1^	Xia et al. (2015)
*T. lanuginosus* SSBP	*tlxyn1*	pBGPI	*P. pastoris* GS115	52.3	Temperature = 50 °C pH = 7 specific activity = 2.29 U mg^−1^	Gramany et al. (2015)

For expression of heterologous proteins, *K. lactis* as a host is known for its applications in food and dairy industries i.e. bovine chymosin. As a platform of recombinant protein expression *K. lactis* furnishes several advantages including applications of both episomal and integrative expression vectors, simple fermentation tools and easy genetic manipulation. d-Xylose and l-arabinose were released by hydrolytic enzymes β-Xylosidase and α-l-arabinofuranosidase, respectively when they were expressed together in *S. cerevisiae* (Margolles-Clark et al. 1996) from *T. reesei* (*H. jecorina*).

With the promoters of alcohol oxidases the methylotrophic yeasts *H. polymorpha* and *P. pastoris* have been successful as expression systems. Alcohol oxidase is the first enzyme of the pathway of methanol utilization and the promoters were named as *AOXI* in *P. pastoris* and *MOX* in *H. polymorpha* (Macauley-Patrick et al. 2005). In *P. pastoris* both glucose and glycerol repress *AOXI* promoter, and in *H. polymorpha* glucose represses *AOXI* promoter while glycerol derepresses *MOX* promoter to about one-fourth of the induced levels. With *AOXI* or *MOX* promoter systems the aerobic growth after induction with methanol resulted in high levels of recombinant proteins (Muller et al. 1998; Voronovsky et al. 2009). Under the control of *AOXI* or *MOX* promoters the multi-copy strains of yeasts with recombinant gene could be the choice for protein expressions at industrial scale. *Y. lipolytica* which is oleaginous yeast is the upcoming protein expression host as it has many applications for heterologous protein expressions. These advantages include its ability to metabolize acetate, glucose, alcohols and hydrophobic substances such as alkanes, oils and fatty acids, resembling mammalian system of glycosylation, well characterized secretory systems yielding high levels of recombinant proteins (i.e. 2 g L^−1^) using defective selection marker, easy screening of multi-copy strains and single integration site usage. The thermotolerant yeast (*H. polymorpha*) was engineered by Voronovsky et al. (2009) coexpressing β-Xylosidase from *A. niger* and endoxylanase from *T. reesei* by the integration of these genes into *H. polymorpha* genome with its promoter glyceraldehyde-3-phosphate dehydrogenase (GAPDH) gene. The resulted transformants were found to be capable of growing and producing ethanol on a minimal medium at 48 °C supplemented with birchwood xylan as a sole carbon source.

### Recombinant β-Xylosidases

The recombinant enzymes are favored over native ones due to certain reasons. In recombinant enzymes we can control production environment as we have choice of different strains and expression vectors for the cloning of an enzymatic system. A more purified product is usually produced from a recombinant enzyme with lesser processing time than native one (Zafar et al. 2014). Usually, fungal β-Xylosidases are active under more acidic conditions i.e. less than pH 5.0, whereas bacterial enzymes have their optimum pHs close to pH 7 (Hayashi et al. 2001). The optimum pH of a recombinant Xyl43B from *Clostridium stercorarium* was found in an acidic pH range and the enzyme was slightly active at pH 7 (Suryani et al. 2004). Moreover, it was also found that the recombinant Xyl43B did not need any metal cofactors as the enzyme activity was not influenced by the addition of various metal ions such as Ba^2+^, Ni^2+^, Fe^2+^, Mg^2+^, Ca^2+^, Mn^2+^ and Zn^2+^ as chloride salts to enzyme assay mixtures. The xylanase activity of Xyl43B was also found much smaller (0.1 U mg^−1^) as compared to other enzyme of this type e.g. XynB (about 4000 U mg^−1^) (Fukumura et al. 1995).

The purification of another recombinant β-Xylosidase (Bxyl) from *B. halodurans* C-125 was facilitated by the presence of *N*-terminal His-tag and the enzyme was found to be highly expressed as it reached about 5 % of total soluble protein (Liang et al. 2009). The specific activity of Bxyl was found 174 mU mg^−1^ of protein that was lower than fungal β-Xylosidases but higher than most of the bacterial xylosidases (Rasmussen et al. 2006). Bxyl was also observed to be very stable because its activity could be maintained for several weeks at 4 °C. Moreover, it is one of the most xylose tolerant enzymes and this quality has made the recombinant enzyme very useful for saccharification of xylan-containing polysaccharides. Suzuki et al. (2010) purified a fungal β-Xylosidase recombinant enzyme XylB from *A. oryzae* with specific activity of 6.1 U mg^−1^ of protein but the enzyme didn’t show any significant xylosidase activity. Unlike other fungal β-Xylosidases, XylB was found to be stable at alkaline pH and it didn’t possess substrate ambiguity. Teng et al. (2011) found that the recombinant enzyme PtXyl43 without predicted signal peptide was secreted in large amount in *E. coli*. Expression of extracellular secretion and efficient production of this enzyme in *E. coli* has increased the importance of PtXy143 for having potential industrial applications.

## Regulation and production of β-Xylosidases

For the production of biofuels, so far the mesophilic engineered organisms or enzymes have been the favored choices. The reasons for this choice are mainly the deep knowledge of their metabolic pathways and genetic tools that have been established to engineer those. But in the last few years, due to stoutness and versatility of thermophilic organisms some alternative approaches have also been made on these organisms and their enzymes (Barnard et al. 2010). It has been observed in most of the fungi that the expression of xylanolytic enzymes is subjected to specific induction in xylan or xylose presence and to carbon catabolite repression that is mediated by the catabolite repressible entities (CreA) repressor (Prathumpai et al. 2004). The transcription activator XlnR also mediates the regulation of β-Xylosidases that regulates the expression of different genes which are involved in the degradation of xylan (Stricker et al. 2008). The transcription and repression of *xlnR* gene is controlled by carbon sources and CreA respectively (Tamayo et al. 2008) therefore, a balance between transcription of XlnR factor and CreA repressor is responsible for transcription regulation of xylanolytic enzymes such as β-Xylosidases. From *T. emersonii* the gene encoding β-Xylosidase (*bxl1*) was isolated and cloned in *E. coli* (Reen et al. 2003). The inferred amino acid sequence reveals homology with β-Xylosidase gene products from *T. reesei*, *A. nidulans* and *A. niger* and with some β-Xylosidase genes belonging to GH family 3. It was found that β-Xylosidase gene was induced by xylan, d-xylose and also by methyl-β-d-xylopyranoside but not with high concentrations. There are six CreA binding sites in the promoter of β-Xylosidase (*bxl1*) gene and the observed repression by d-glucose was suggested to be mediated by this catabolite repressor. To regulate and secrete inducible enzymes the catabolic repression plays a vital role. The catabolic repression of β-Xylosidases at molecular level has been related with the presence of CreA binding sites in their promoters and this carbon catabolite repression that is mediated by CreA has also been reported in other fungal genes (Knob et al. 2010).

The induction of mutations in the parental fungal strains using various mutagens has been the most efficient practice for enhanced productions of important enzymes (Mustafa et al. 2014). Increased levels of regulatory proteins and co-factors of different fungal mutants are considered responsible for enhanced activities of β-Xylosidases. Maximum production of β-Xylosidase was achieved (728 IUg^−1^ substrate, Y_P/S_) from *H. lanuginosa* M7D mutant grown on Vogel’s medium containing xylan. *K*_*m*_ value for purified mutant enzyme was 1.8 mm with optimum pH 8.5 and temperature of 60 °C (Bokhari et al. 2010). In another study, maximum volumetric production of β-Xylosidase from *C. biazotea* mutant grown on xylan was achieved 30.7 IU/l/h which was 2.29-fold enhanced over its wild strain (Rajoka et al. 1997). An enhanced production of β-Xylosidase was also achieved from *K marxianus* M125 mutant up to 1.5 to twofold more than that from the wild strain. The stability of the purified enzyme was found good at a temperature of 60 °C and pH 5.0–7.0. The results suggested that an induction mechanism was involved in the regulation of β-Xylosidase biosynthesis which enhanced specific yield of enzyme (Y_p/x_) up to 59-fold in the mutant cells (Rajoka and Khan 2005). To regulate and secrete inducible enzymes the catabolite repression plays a vital role which has also been studied in *K. marxianus* var. *marxianus*. Due to mixed inductive or repressive effects an increased production of β-Xylosidase in xylose medium with glucose was observed (6.8 IU mL^−1^) in *K. marxianus* var. *marxianus* (Rajoka 2007). When regulation of β-Xylosidase production was studied in *Cellulomonas flavigena*, maximum yield of the enzyme was obtained with xylose (monomeric sugar) in Dubos medium. Cellobiose was observed as best inducer and xylan as best substrate of β-Xylosidase among disaccharides and polymeric substances respectively (Rajoka 2005).

In general, during β-Xylosidase productions the monosaccharides play roles as strong repressors while disaccharides and polysaccharides act as inducers. During the growth of β-Xylosidase on monosaccharides a very low quantity of mRNA is produced for enzyme and the expression of enzyme is modulated by CreA on xylose or glucose in filamentous fungi. The efficiency of β-Xylosidase inducers can be checked through their binding affinity with those of regulatory macromolecules and their actual concentration inside the cell.

## Coding regions of β-Xylosidase gene

β-Xylosidases belong to glycosyl hydrolases families 3, 39, 43, 51, 52 and 54, but so far the fungal enzymes are described only from families 3, 43 and 54. In databases s2 is the only β-Xylosidase gene which has been completely sequenced from *Penicillium herquei* IFO 4674. The gene consists of 1005 base pairs and does not contain any introns. Although, the enzyme is a cell surface associated protein but it encodes 335 amino acid protein without any apparent signal peptide (Ito et al. 2003). In addition to this sequence, a partial gene sequence of β-Xylosidase of 290 bp has also been reported from *P. purpurogenum* (Ito et al. 2003). A high degree of similarity is possessed by these both enzymes with glycosyl hydrolases family 43. It is an interesting observation that from organisms with phylogenetic closeness with *Penicillium* such as *Aspergillus* are not found in family 43 that contains mainly xylosidases from bacterial origins but found in glycosyl hydrolases family 3. However, the open reading frames (ORFs) from some fungi whose entire genome has been sequenced encode for hypothetical proteins having similarity with *Penicillium* β-Xylosidases described above. β-Xylosidase genes from *A. nidulans* (accession number XP 405614 in NCBI Protein Database), *Magnaphorte grisea* (accession number XP 366835 in NCBI Protein Database) and *Neurospora crassa* (Galagan et al. 2003) has been isolated and sequenced. In addition, a partial sequence of β-Xylosidase from *A. oryzae* (Ito et al. 2003) and from *Cochliobolus carbonum* β-Xylosidase (Wegener et al. 1999) also belong to family 43. It has been suggested from these findings that fungal β-Xylosidases are structurally of two types and are categorized into two different families of glycosyl hydrolases i.e. 3 and 43.

## Comparisons of amino acid sequences of fungal β-Xylosidases

**Fig. 2:**
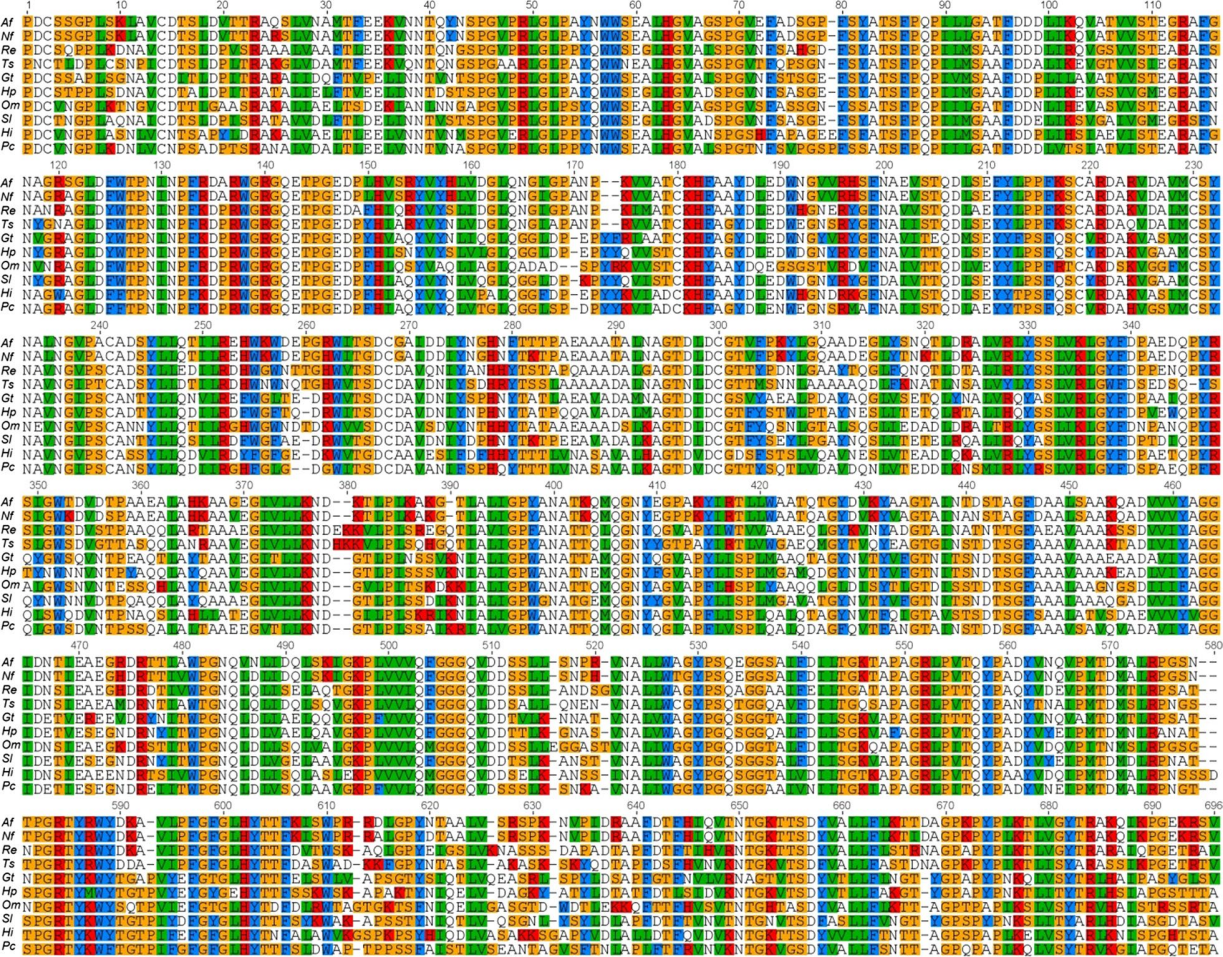
Alignment of β-Xylosidase enzymes from ten fungal species. Xylosidases are from are from *Aspergillus fumigatus* (*Af*), *Neosartorya fischeri* (*Nf*), *Rasamsonia emersonii* (*Re*), *Talaromyces stipitatus* (*Ts*), *Gloeophyllum trabeum* (*Gt*), *Hydnomerulius pinastri* (*Hp*), *Oidiodendron maius* (*Om*), *Serpula lacrymans* (*Sl*), *Heterobasidion irregulare* (*Hi*), *Phanerochaete carnosa* (*Pc*)

A multiple sequence alignment was performed to compare sequences of β-Xylosidases from ten different fungal species (Fig. 2). The amino acid sequence of β-Xylosidase from *A. fumigatus* was aligned and compared with β-Xylosidases from nine different fungal species. It showed β-Xylosidase similarities of 95 % from *Neosartorya fischeri*, 70 % from *Rasamsonia emersonii*, 69 % from *Talaromyces stipitatus*, 60 % from *Gloeophyllum trabeum*, 59 % from *Hydnomerulius pinastri*, 58 % from *Oidiodendron maius*, 58 % from *Serpula lacrymans*, 58 % from *Heterobasidion irregular* and 59 % from *Phanerochaete carnosa*.

Through hydrophobic cluster analysis many β-Xylosidases have been classified into three different families i.e. 39, 43 and 52 (Henrissat and Bairoch 1993) and among these three distinct groups of β-Xylosidases, *XylA* (a β-Xylosidase gene from *A. oryzae*) gene did not show any similarity to these groups. However, *XylA* gene showed 70 % similarities with *XlnD* from *A. nidulans*, 64 % with *XlnD* from *A. niger* and 63 % with *BxlI* from *T. reesei.* The sequence similarities have shown that all these fungal β-Xylosidase genes belong to family 3 glycoside hydrolase (GH) (Kitamoto et al. 1999). Moreover, *A. oryzae* has also been reported to have two cell wall-binding and three extracellular types of β-Xylosidases (Hashimoto et al. 1999). Several highly conserved regions among *XylA* of *A. oryzae*, *XlnD* of *A. nidulans*, *BxlI* of *T. reesei* and *XlnD* of *A. niger* have also been found that have been hypothesized to be involved in substrate binding, catalytic reactions or both (Kitamoto et al. 1999). An Asp residue of one putative active-site has been thought to be playing a role in catalytic activity as determined for β-glucosidase A3 from *A. wentii* and it was also found to be conserved in *XylA* of *A. oryzae* (Asp-310) (Bause and Legler 1980).

A β-Xylosidase *XylA* was also reported from *A. japonicus* and its BLAST search gave high degrees of similarities with other β-Xylosidases of GH family-3 (Wakiyama et al. 2008). It showed 69 % sequence similarity with *XlnD* from *A. niger* (van Peij et al. 1997), 69 % with *Xawl* from *A. awamori* (Kurakake et al. 2005), 69 % with *XylA* from *A. fumigatus* (CM000169; locus tag AFUA_1G16920), 68 % with *XylA* from *A. oryzae* (Kitamoto et al. 1999), 66 % with *Bxl1* from *T. emersonii* (Reen et al. 2003), 65 % with *XlnD* from *A. nidulans* (Perez-Gonzalez et al. 1998), 64 % with *Bxl1* from *T. reesei* (Margolles-Clark et al. 1996), 63 % with β-Xylosidase from *A. clavatus* and 48 % with β-Xylosidase from *N. fischeri* (DS027697; locus tag NFIA_003180).

A BLAST analysis for another β-Xylosidase *Xyp1* from a plant-pathogenic fungus *C. carbonurn* yielded some surprising results as it did not show any similarity with any of fungal β-Xylosidases present in database (Altschul et al. 1990) but it showed identity with regard to its size and primary sequence with two bacterial β-Xylosidases i.e. 30 % overall identity each with *Bacteroides ovatus* and *Prevotella ruminicola* (Whitehead and Hespell 1990; Gasparic et al. 1995). Surprisingly, no obvious signal peptide was present in any of these two bacterial xylosidases therefore, *P. ruminicola* β-Xylosidase was supposed to be present in cytoplasm (Gasparic et al. 1995).

Reen et al. (2003) compared the deduced amino acid sequence of *bxl1* gene encoding β-Xylosidase from a thermophilic fungus *T. emersonii* and compared with the sequences available in databases. The sequence of *bxl1* showed identities with β-Xylosidase genes following 73 % from *T. reesei*, 64 % from *A. oryzae*, 63 % with *A. nidulans* and 61 % with *A. niger*. Homolog of *bxl1* gene with β-glucosidases of GH family-3 was also observed with identity values of 38 % from *Xanthomonas campestris*, 33 % from *Bacillus halodurans* and 22 % from *T. Reesei* with Asp311 appeared in majority of β-glucosidases of GH family-3 as conserved residue and suggested that it is a part of active site of β-glucosidases of GH family-3 (Coutinho and Henrissat 1999).

Surprisingly, Margolles-Clark et al. (1996) reported that amino acid sequence of *BXLI* gene from *T. reesei* didn’t show similarities with any reported β-Xylosidases from the families of 39, 43 and 52. In spite of lacking the β-glucosidase activity the gene showed significant similarities with family 3 enzymes and this family is highly conserved as it includes β-glucosidases only. Another β-Xylosidase gene (*Xawl*) was deduced from *A. awamori* and searched for its homology (Kurakake et al. 2005). The amino acid sequence of the gene showed similarities with β-Xylosidases from *A. niger* having 98 % (van Peij et al. 1997) and 94 % (Grange et al. 2000) identities with DNA sequence.

A novel β-Xylosidase gene (*PtXyl43*) was isolated from thermophilic fungus *Paecilomyces thermophile*, cloned and studied for its homology (Teng et al. 2011). The gene showed high identities with some putative fungal β-Xylosidases of GH family-43. It gave similarities with β-Xylosidases from *Penicillium herquei* 73 % (Ito et al. 2003), *A. fumigatus* 72 %, *N. fischeri* 72 %, *Penicillium chrysogenum* 71 %, *A. flavus* 70 % and *A. oryzae* 70 %.

Currently, β-Xylosidases from different fungi have been classified into five glycosyl hydrolase (GH) families i.e. 3, 39, 43, 52 and 54. Most of the β-Xylosidases from fungi have shown high sequence similarities with other fungal β-Xylosidases but *Xyp1* from a plant-pathogenic fungus *Cochliobolus carbonurn* did not show similarity with fungal β-Xylosidases and it showed identity with bacterial β-Xylosidases of *B. ouatus* and *P. ruminicola*.

## Concluding remarks

Degradation of a complex mixture of biomass is an essential evil for the production of easily accessible sugar residues in industrial processes. Enhanced production of industrial biocatalysts with novel and desirable characteristics have better prospects in terms of both increased economic pressure and industrial developments. Over-production of β-Xylosidase could not receive much attention due to limited information regarding crystallographic structure of the enzyme; however recombinant enzyme with pH and temperature stability is a good alternative to the native enzyme for its effective utilization. Despite great hopes placed in biotechnological advances, hyperproduction of enzymes is still challenging. Although heterologous expression of β-Xylosidase in hosts such as *E. coli*, *P. pastoris* and *S. cerevisiae* is attractive due to less interfering activities, glycosylation and post translational modification complicate the issue.

Exploring new fungal hosts capable of producing recombinant β-Xylosidases is still a possibility. To improve and develop fungal expression systems using approaches of genetic engineering through further technical advancements will surely help in hyperexpression of heterologous β-Xylosidases from different fungi. Hyperproduction of the enzyme can be achieved by conventional mutagenesis of the strains or by using knockout strains as a host. Homologous expression can also be an alternative but with major problem of contamination of endogenous hydrolases. Contamination can be avoided by using His tags engineered into a protein for purification. Thus *P. pastoris* and *S. cerevisiae* can be a reasonable host for medium scale production of β-Xylosidase.

β-Xylosidases have high amino acid sequence similarities from different fungi. Several β-Xylosidases have been purified from bacteria and fungi, and studied for their biochemical characterization. In general, based on their amino acid sequence similarities, they are classified into five different families of glycosyl hydrolases (GH) which include GH families 3, 39, 43, 52 and 54. Most of the reported fungal enzymes belong to GH family-3 of β-Xylosidases in spite of they have been reported from other GH families as well. Sequence similarities searches of β-Xylosidases on databases play an important role to identify and classify some novel β-Xylosidase genes from different fungi.
